# Uniquely Satisfiable *d*-Regular (*k*,*s*)-SAT Instances

**DOI:** 10.3390/e22050569

**Published:** 2020-05-19

**Authors:** Zufeng Fu, Daoyun Xu

**Affiliations:** 1 College of Computer Science and Technology, Guizhou University, Guiyang 550025, China; fuzufeng@outlook.com; 2 Department of Electronics and Information Engineering, Anshun University, Anshun 561000, China

**Keywords:** *d*-regular (*k*,*s*)-CNF, SAT-problem, uniquely satisfiable

## Abstract

Unique *k*-SAT is the promised version of *k*-SAT where the given formula has 0 or 1 solution and is proved to be as difficult as the general *k*-SAT. For any k≥3, s≥f(k,d) and (s+d)/2>k−1, a parsimonious reduction from *k*-CNF to *d*-regular (*k*,*s*)-CNF is given. Here regular (*k*,*s*)-CNF is a subclass of CNF, where each clause of the formula has exactly *k* distinct variables, and each variable occurs in exactly *s* clauses. A *d*-regular (*k*,*s*)-CNF formula is a regular (*k*,*s*)-CNF formula, in which the absolute value of the difference between positive and negative occurrences of every variable is at most a nonnegative integer *d*. We prove that for all k≥3, f(k,d)≤u(k,d)+1 and f(k,d+1)≤u(k,d). The critical function f(k,d) is the maximal value of *s*, such that every *d*-regular (*k*,*s*)-CNF formula is satisfiable. In this study, u(k,d) denotes the minimal value of *s* such that there exists a uniquely satisfiable *d*-regular (*k*,*s*)-CNF formula. We further show that for s≥f(k,d)+1 and (s+d)/2>k−1, there exists a uniquely satisfiable *d*-regular (k,s+1)-CNF formula. Moreover, for k≥7, we have that u(k,d)≤f(k,d)+1.

## 1. Introduction

Satisfiability Problem (SAT) is a central problem in theoretical computer science of deciding whether a given Conjunction Normal Formula (CNF) is satisfiable. The *k*-SAT is a satisfiability problem where every clause has exactly *k* distinct variables, and was proved to be a NP-complete problem for k≥3 in [1]. That is, SAT problem should be a computationally hard problem. However, modern SAT solvers are able to efficiently solve some formulas with millions of variables, such as MiniSat [2], Glucose [3], Maple [4]. The conflict-driven clause learning technique is an important algorithm to improve the efficiency of these SAT solver. Yet, how these solvers can be so successful has remained elusive. In order to analyze and improve SAT solvers, some random SAT models were propose.

A natural measure of the solution space is the number of solutions. Unique *k*-SAT denotes the promise search problem of *k*-SAT where the number of solutions is either 0 or 1. The harder instances should have fewer solutions. But Calabro and Paturi in [5] proved that the exponential complexity of deciding whether a *k*-CNF formula has a solution is the same as that of deciding whether it has exactly one solution, both when it is promised and when it is not promised that the input formula has a solution. Thus, the research of uniquely satisfiable SAT instances is a very significant work.

The (k,s)-SAT denotes the family of satisfiability problems restricted to CNF formulas with exactly *k* distinct variables per clause and at most *s* occurrences of each variable. Regular (k,s)-SAT is a class of special (k,s)-SAT which each variable occurs in exactly *s* clauses. By some polynomial time reductions, it is discovered that some SAT problems with regular structures are NP-complete, such as (3,4)-SAT problem in [6] and regular (3,4)-SAT problem in [7]. Experimental results and theoretical analysis on a random *k*-SAT problem showed that the constrained density α of a CNF formula is an important parameter affecting the formula satisfiability and the solving difficulty in [8,9,10,11]. There is a phase transition point α(k) on a random *k*-SAT problem such that

(i) all random *k*-CNF instances with α<α(k) are satisfiable with high probability;
(ii) all random *k*-CNF instances with α>α(k) are unsatisfiable with high probability.

But every regular (k,s)-CNF formula has a fixed constrained density α (the clause-to-variable ratio), such as regular (3,4)-CNF formula corresponding to 4/3. The constrained density of the regular (3,4)-CNF is much smaller than the SAT-UNSAT phase transition point of the random 3-SAT problem α(3)≈4.267 in [12]. This shows that a random regular (3,4)-CNF formula is satisfiable with high probability, but the regular (3,4)-SAT problem is NP-complete. Obviously, it is not enough to describe structural features of the CNF formula merely by the constrained density α.

In [13,14], M. Wahlström presented a definition of (a,b)-variable to classify all variables in a CNF formula, and designed two algorithms for solving a CNF formula with at most *d* occurrences per variable. Here, an (a,b)-variable is a variable which occurs positively in *a* clauses and negatively in *b* clauses. In [15], Johannsen, Razgon and Wahlström presented an algorithm for solving a CNF formula in which the number of occurrences of each literal is at most *d*. Their results demonstrated that the CNF formulas with some restrictions on the number of occurrences (positive or negative) of each variable have its own characteristics.

In order to further study SAT problems with regular structures, we introduced *d*-regular (k,s)-CNF formula in [16,17]. The regular (k,s)-CNF formula requires that each clause contains exactly *k* variables and each variable occurs in exactly *s* clauses. The *d*-regular (k,s)-CNF formula also requires that the absolute value of the difference between positive and negative occurrences of each variable is no more than a nonnegative integer *d*. In this paper, we investigate the existence condition of uniquely satisfiable *d*-regular (k,s)-SAT Instances, and present a method to construct a uniquely satisfiable *d*-regular (k,s)-formula. We also give a parsimonious reduction from *k*-CNF to *d*-regular (k,s)-CNF, and further explain the constrained density is not enough to describe the structural features of a CNF formula.

## 2. Related Works

Unique SAT is the promised version of the SAT, where a given CNF formula has 0 or 1 solution. Valiant and Vazirani in [18] gave a randomized polynomial time reduction from SAT to Unique SAT, and showed that deciding whether a CNF formula has zero or one solution is essentially as difficult as SAT in general. Calabro et al. in [19] proved that Unique *k*-SAT is no easier than *k*-SAT, not just for polynomial time algorithms but also super-polynomial time algorithms. They in [5] pointed out it does not matter whether there has a promise that a formula has a solution. Matthews in [20] studied the complexity of UNIQUE-(k,s)-SAT and proved that f(k)≤u(k)≤f(k)+2 for k≥3, where u(k) is the minimal value of *s* so that uniquely satisfiable (k,s)-CNF formulas exist and f(k) represents the maximal value of *s* such that all (k,s)-CNF formulas are satisfiable. The exact values of f(k) are only known for k=3 and k=4, because f(3)=3, f(4)=4 were shown in [21]. In [22,23,24,25], it showed that the upper and lower bounds for k=5,6,…,9, f(k) are described as follows

5≤f(5)≤7,7≤f(6)≤11,13≤f(7)≤17,24≤f(8)≤29,41≤f(9)≤51.

Encoding into a CNF formula is a common way to solve a practical problem. These CNF formulas often have some special structures and properties. It is important to design some random SAT models that are similar to reality. Markström in [26] proposed a constructor method of SAT instance based on Eulerian graphs, and discussed how a solver can try to avoid at least some of the pitfalls presented by these instances. Giraldez-Cru and Levy in [27] proposed a new model of generation of random SAT instances with community structure, and showed that modern solvers do actually exploit this community structure. In [28], they presented a random SAT instances generator based on the notion of locality, and showed that CDCL SAT solvers take advantage of both popularity and similarity. In [29,30], it showed that SAT instances with less solutions tend to be harder for stochastic local search methods. In [31], Žnidarič gave an experimental evaluation of uniquely satisfiable 3-SAT instances obtained by simply filtering randomly generated formulas.

In this paper, we investigate a uniquely satisfiable *d*-regular (k,s)-SAT Instances, and show that f(k,d)≤u(k,d)+1, f(k,d+1)≤u(k,d) for k≥3, and u(k,d)≤f(k,d)+1 for k≥7. Here u(k,d) denotes the minimal value of *s* such that uniquely satisfiable *d*-regular (k,s)-CNF formulas exist, and f(k,d) denotes the maximal value of *s* such that all *d*-regular (k,s)-CNF formulas are satisfiable. We demonstrate that for s≥f(k,d)+1 and (s+d)/2>k−1, there is a uniquely satisfiable *d*-regular (k,s)-CNF formula. We also reveal that for k≥7, if a *d*-regular (k,s)-CNF formula is unsatisfiable, then (s+d)/2>k−1. Finally, for k≥3, s≥f(k)+1 and (s+d)/2>k−1, we give a parsimonious reduction from a *k*-CNF formula to a *d*-regular (k,s)-CNF formula. Constructing uniquely satisfiable *d*-regular (k,s+1)-CNF formulas from an unsatisfiable *d*-regular (k,s)-CNF formula is a key component of our reduction.

## 3. Notations

A literal is a boolean variable *x* or a negated boolean variable ¬x. *x* is called a positive literal, and ¬x is called a negative literal. A clause *C* is a disjunction of literals, $C=L_{1}\vee L_{2}\vee \ldots \vee L_{k}$ or $C=\{L_{1},L_{2},\ldots ,L_{k}\}$. A formula *F* in the conjunctive normal formula is a conjunction of clauses, $F=C_{1}\wedge C_{2}\wedge \ldots \wedge C_{m}$ or $F=\{C_{1},C_{2},\ldots ,C_{m}\}$. var(F) denotes the set of boolean variables occurring in a formula *F*, and #var(F) refers to the number of variables occurring in *F*. #cl(F) denotes the number of clauses of *F*, and pos(F,x) (neg(F,x)) refers to the number of positive (negative) occurrences of a variable *x* in *F*. pos(F) (neg(F)) denotes the number of positive (negative) literals in *F*, and pos(F,X) (neg(F,X)) refers to the number of positive (negative) occurrences of all variables of the variable set *X* in *F*.

A truth assignment τ is a function which assigns to each boolean variable *v* a unique value τ(v)={0,1}. A CNF formula *F* is satisfiable, if a truth assignment τ with τ(F)=1 exists. Such a truth assignment is called a satisfying assignment. We divide boolean variables in these formulas into forced variables or unforced variables. If every satisfying assignment of a formula sets a variable to the same value, we call it a forced variable. Otherwise, the variable is regarded as an unforced variable.

If the formulas Φ and Ψ are either satisfiable at the same time or not, they are called SAT-equivalents. This implies that, Φ is satisfiable if and only if Ψ is satisfiable. A formula $F^{\prime }$ is called the disjoint copy of a CNF formula *F*, if $F^{\prime }$ is a copy of *F* and their variable sets are disjoint. A uniquely satisfiable *d*-regular (k,s)-CNF formula is a *d*-regular (k,s)-CNF with only one solution. A CNF formula *F* is a minimal unsatisfiable formula (MU), if *F* is unsatisfiable and F−{C} is satisfiable for any clause C∈F. For a given unsatisfiable formula *F*, a minimal unsatisfiable formula can be obtained by removing some clauses from *F*.

**Definition** **1.** *For each k≥3, f(k) is defined as the maximal value of s such that all (k,s)-CNF formulas are satisfiable, f(k,d) is defined as the maximal value of s such that all d-regular (k,s)-CNF formulas are satisfiable, u(k) is defined as the minimal value of s such that uniquely satisfiable (k,s)-CNF formulas exist, and u(k,d) is defined as the minimal value of s such that uniquely satisfiable d-regular (k,s)-CNF formulas exist.*

**Definition** **2.** *A k-CNF formula F is called a k-forced-once d-regular (k,s)-CNF formula if*

*(i)*  *there exist k variables $x_{1},x_{2},\ldots ,x_{k}$ that only occur once;*
*(ii)*  *except for the k variables, every variable occurs in exactly s clauses, and the absolute value of the difference between positive and negative occurrences of every variable is no more than the nonnegative integer d.*
*(iii)*  *F is satisfiable and for any truth assignment τ satisfying F, it holds that*
  $$\tau \left(x_{1}\right)=\tau \left(x_{2}\right)=\ldots =\tau \left(x_{k}\right)=true.$$

We can represent a CNF formula as a matrix. Each variable $x_{i}$ corresponds to a row of the matrix and each clause $C_{j}$ corresponds to a column of the matrix. For each variable $x_{i}$, if its positive (resp., negative) literal is in the clause $C_{j}$, then $a_{i,j}=+$ (resp., $a_{i,j}=-$); otherwise, 0.

Let *F* is a CNF formula with 15 variables $x_{1},x_{2},\ldots ,x_{15}$ and 25 clauses $C_{1},C_{2},\ldots ,C_{25}$. The representation matrix of the formula *F* is

$$\begin{array}{c} x_{1}\\ x_{2}\\ x_{3}\\ x_{4}\\ x_{5}\\ x_{6}\\ x_{7}\\ x_{8}\\ x_{9}\\ x_{10}\\ x_{11}\\ x_{12}\\ x_{13}\\ x_{14}\\ x_{15} \end{array}\left(\begin{array}{ccccccccccccccccccccccccc} + & - & + & + & & & - & - & & & & & & & & & & & & & & & & & \\ + & - & - & - & + & + & & & & & & & & & & & & & & & & & & & \\ + & - & & & - & - & + & + & & & & & & & & & & & & & & & & & \\ & & + & - & + & - & + & & & & & & & & & & & & & & & & & & -\\ & & & & & & & + & & & & & & & & & - & - & - & + & + & & & & \\ & & & & & & & & + & - & + & + & & & - & - & & & & & & & & & \\ & & & & & & & & + & - & - & - & + & + & & & & & & & & & & & \\ & & & & & & & & + & - & & & - & - & + & + & & & & & & & & & \\ & & & & & & & & & & + & - & + & - & + & & & & & & & & & - & \\ & & & & & & & & & & & & & & & + & - & - & - & & & + & + & & \\ & & & & & & & & & & & & & & & & & & & - & - & - & + & + & +\\ & & & & & & & & & & & & & & & & & & & + & + & + & - & - & -\\ & & & & & & & & & & & & & & & & + & & & & & & & & \\ & & & & & & & & & & & & & & & & & + & & & & & & & \\ & & & & & & & & & & & & & & & & & & + & & & & & & \end{array}\right).$$

Clearly, *F* is a 3-forced 0-regular (3,6)-CNF formula. Each of the three variables $x_{13},x_{14},x_{15}$ occurs in exactly one clause in *F* and is forced to be true.

**Definition** **3.** *In the context of SAT, a reduction M is identified to be parsimonious if x and M(x) have the same number of satisfying assignments for any one formula x.*

**Lemma** **1** ([32])**.** *Let (k,s)-CNF be a class of satisfiable formulas, then all (k+r,s+r[s/k])-CNF formulas are satisfiable for any nonnegative integer r ([x] denotes the integral part of x).*

**Lemma** **2** ([17])**.** *If the representation matrix of a formula F is*

$$\begin{array}{c} x_{1}\\ x_{2}\\ \vdots \\ \vdots \\ x_{n-1}\\ x_{n} \end{array}\left(\begin{array}{ccccc} + & \; & \; & \; & -\\ - & + & \; & \; & \;\\ \; & - & \; & \; & \;\\ \; & \; & \ddots & \; & \;\\ \; & \; & \; & + & \;\\ \; & \; & \; & - & + \end{array}\right),$$

*then the formula is satisfiable and every satisfying assignment forces all variables to a same value.*

## 4. Uniquely Satisfiable *d*-Regular (k,s)-CNF Formula

The *d*-regular (k,s)-CNF formula has stronger regular constraints than the regular (k,s)-CNF formula. It limits the absolute value of the difference between positive and negative occurrences of each variable. The uniquely satisfiable *d*-regular (k,s)-CNF formula refers to a *d*-regular (k,s)-CNF formula with only one solution. We investigate the existence conditions of the uniquely satisfiable *d*-regular (k,s)-CNF formula.

**Theorem** **1.** *For all k≥3, f(k,d)≤u(k,d)+1 and f(k,d+1)≤u(k,d).*

**Proof.**  Because f(k,d) denotes the maximal value of *s* such that all *d*-regular (k,s)-CNF formulas are satisfiable, we usually construct an unsatisfiable *d*-regular (k,s)-CNF formula to find the upper bound of f(k,d).

Let s=u(k,d). Because u(k,d) denotes the minimal value of *s* such that uniquely satisfiable *d*-regular (k,s)-CNF formulas exist, there must be a uniquely satisfiable *d*-regular (k,s)-CNF formula *F*. Obviously, by adding a clause to *F* which is violated by the unique satisfying assignment, the formula *F* can become an unsatisfiable formula. Suppose the formula *F* has *n* variables. We give two methods to construct unsatisfiable instances.

Method 1: We introduce ⌈n/k⌉k−n new variables and add ⌈n/k⌉(s+2)−ns/k new clauses to *F*, which contains at least one clause violated by the unique satisfying assignment. Let each original variable occurs twice in the new clauses (one negative occurrence and another positive occurrence), and each new variable occurs s+2 times in the new clauses (the number of positive and negative occurrences of every new variable is nearly equal). That is, each variable occurs s+2 times in *F* and the absolute value of the difference between positive and negative occurrences of each variable is no more than *d*. Therefor, *F* is turned into an unsatisfiable *d*-regular (k,s+2)-CNF formula. It can be seen that f(k,d)≤s+1=u(k,d)+1.

Method 2: We introduce ⌈n/k⌉k−n new variables and add ⌈n/k⌉(s+1)−ns/k new clauses to *F*, which contains at least one clause violated by the unique satisfying assignment. Let each original variable occur once in the new clauses, and each new variable occurs s+1 times in the new clauses (the number of positive and negative occurrences of every new variable is nearly equal). That is, each variable occurs s+1 times in *F* and the absolute value of the difference between positive and negative occurrences of each variable is no more than d+1. Therefor, *F* is turned into an unsatisfiable (d+1)-regular (k,s+1)-CNF formula. It can be seen that f(k,d+1)≤s=u(k,d). □

**Lemma** **3.** *If k≥3 and s are two nonnegative integers such that an unsatisfiable d-regular (k,s)-CNF formula exists, there exists a k-forced-once d-regular (k,s)-CNF formula.*

**Proof.**  Let Φ be an unsatisfiable *d*-regular (k,s)-CNF formula. Obviously, the number of positive occurrences and negative occurrences of every variable in Φ are all no more than (s+d)/2. By removing some clauses of Φ, a minimal unsatisfiable (k,s)-CNF formula $\Phi_{1}$ can be obtained. It is easy to get that, the number of positive occurrences and negative occurrences of every variable in $\Phi_{1}$ are all no more than (s+d)/2.

Let $\Phi =\Phi_{1}\wedge \Phi_{2}$, where $\Phi_{1}$ is the unsatisfiable (k,s)-CNF formula obtained by removing some clauses of Φ, and $\Phi_{2}$ is a conjunction of the removed clauses. Suppose $\Phi_{2}$ contains m≥0 clauses and mk literals. Let $C_{1}$ be the clause set of $\Phi_{1}$ and $C_{2}$ be the clause set of $\Phi_{2}$. A variable *y* of $var(\Phi_{1})$ and a clause *c* containing ¬y are randomly selected. Define $C=\left(C1\setminus \left\{c\right\}\right)\cup \left\{\tilde{c}\right\}$, with $\tilde{c}=\left(c\setminus \left\{\neg y\right\}\right)\cup \left\{x\right\}$, where *x* is a new extra variable that does not occur in Φ. Define $\Phi_{1}^{\prime }=\wedge_{c\in C}c$. Clearly, the variable *x* is forced to be true.

Let $\Phi_{1i}$ be disjoint copies of the formula $\Phi_{1}^{\prime }$ with the variable x,y of $\Phi_{1}^{\prime }$ being renamed as $x_{i},y_{i}$ in $\Phi_{1i}$, and $\Phi_{2i}$ be disjoint copies of the formula $\Phi_{2}$, for 1≤i≤k. In addition, we ensure that every variable occurring both in $\Phi_{1}^{\prime }$ and $\Phi_{2}$ is renamed as a same new variable in $\Phi_{1i}$ and $\Phi_{2i}$, respectively, for 1≤i≤k.

Introduce a new boolean variable set $Z=z_{1},z_{2},\ldots ,z_{tk}$ which does not occur in Φ, t>2mk/s. The *k*-CNF formula $\Phi_{3}$ is constructed using $\neg y_{i}$, the literals of $\Phi_{2i}$ and the variables of *Z*, for 1≤i≤k. And it shall meet the following limits.

(i) Every variable of *Z* occurs positively in ⌈s/2⌉ clauses and negatively in ⌊s/2⌋ clauses;
(ii) All literals of $\Phi_{2i}$ and $\neg y_{i}$ occur exactly once in $\Phi_{3}$, 1≤i≤k;
(iii) Every clause of $\Phi_{3}$ must have at least one positive occurrence of any one of *Z*.

Define $\Phi^{\prime }=\Phi_{3}\wedge \Phi_{11}\wedge \Phi_{12}\wedge \ldots \wedge \Phi_{1k}$.

Obviously, condition (i) and (ii) of Definition 2 hold in $\Phi^{\prime }$ (note s>3 from the unsatisfiability of Φ). $\Phi_{1i}$ is satisfiable and forces the variable $x_{i}$ to be true. Because every variable of *Z* does not occur in $\Phi_{1}^{\prime }$, $\Phi_{3}$ is satisfiable (let the value of every variable of *Z* be true) without affecting $\Phi_{11},\Phi_{12},\ldots ,\Phi_{1k}$. So it can be concluded that $\Phi^{\prime }$ is satisfiable and forces $x_{1},x_{2},\ldots ,x_{k}$ to be true. $\Phi_{1}^{\prime }$, $\Phi_{2}$ and ¬y only contain *x* and all literals of Φ. Except for *x*, every variable of $\Phi_{1}^{\prime }$, $\Phi_{2}$ and ¬y occur in *s* clause, and meet the *d*-regularity (Φ is a *d*-regular (k,s)-CNF formula). Hence, $\Phi_{1i}^{\prime }$, $\Phi_{2i}$ and $\neg y_{i}$ meet these requirements (by the definition of disjoint copy). Every variable of *Z* occurs positively in ⌈s/2⌉ clauses and negatively in ⌊s/2⌋ clauses. Thus, $x_{1},x_{2},\ldots ,x_{k}$ occur only once in $\Phi^{\prime }$. Except for the *k* variables, every variable occurs in exactly *s* clauses, and the absolute value of the difference between positive and negative occurrences of every variable is no more than *d*. Therefore, we claim that $\Phi^{\prime }$ is a *k*-forced-once *d*-regular (k,s)-CNF formula.

Next, we will assess the feasibility of the construction of $\Phi^{\prime }$. If an unsatisfiable *d*-regular (k,s)-CNF formula Φ exists, $\Phi_{1}^{\prime }$ should be easily constructed. The number of literals of $\Phi_{3}$ is $mk^{2}+tks+k$, and the number of positive occurrences of the variables of *Z* in $\Phi_{3}$ is kt⌈s/2⌉. The number of clauses of $\Phi_{3}$ is mk+ts+1. For t>2mk/s, we obtain mk<ts/2, mk+ts<3ts/2. For k≥3, we obtain mk+ts+1≤kts/2≤kt⌈s/2⌉. As a result, the number of positive occurrences of *Z* in $\Phi_{3}$ is greater than that of clauses of $\Phi_{3}$. The construction of $\Phi_{3}$ is almost random (First let each clause get a positive literal of *Z*, then randomly arrange other literals). Therefore, $\Phi_{3}$ can be constructed in polynomial time. □

**Lemma** **4.** *For k≥3, (s+d)/2>k−1 and m≥1, we can transform a k-forced-once d-regular (k,s)-CNF formula with n unforced variables into a (m+1)k-forced-once d-regular (k,s)-CNF formula with n unforced variables.*

**Proof.**  Let Φ be a *k*-forced-once *d*-regular (k,s)-CNF formula with *n* unforced variables, and $x_{1},x_{2},\ldots ,x_{k}$ denote *k* forced variables that only occur once. That is, $x_{1},x_{2},\ldots ,x_{k}$ are forced to be true. Let

$$\begin{array}{cc} H_{0} & =\wedge_{i=1}^{k}\left(\neg x_{1}\vee \neg x_{2}\vee \ldots \vee \neg x_{k-1}\vee y_{1,i}\right),\\ H_{j} & =\wedge_{i=1}^{k}\left(\neg y_{j,1}\vee \neg y_{j,2}\vee \ldots \vee \neg y_{j,k-1}\vee y_{j+1,i}\right),j=1,2,\ldots ,mk-1, \end{array}$$

where every $y_{j,i}$ is a fresh variable.

We construct a *k*-CNF formula Ψ with the variable set $X=\{x_{i}\}$ and the variable set $Y=\{y_{j,i}\}$, for i=1,2,…,k−1, j=1,2,…,mk−1, which meets the following restrictions.

(i) every variable of *X* and *Y* occurs in exactly s−k−1 clauses of Ψ,
  $$\mathrm{if}s\leq 2k,pos\left(\Psi ,x_{i}\right)=s-k-1,pos\left(\Psi ,y_{j,i}\right)=s-k-1;$$
  $$\mathrm{if}s>2k,pos\left(\Psi ,x_{i}\right)=\left\lceil s/2\right\rceil -1,pos\left(\Psi ,y_{j,i}\right)=\left\lceil s/2\right\rceil -1.$$
(ii) Every clause of Ψ must have at least one positive occurrence of any one of these variables.

Define $\Phi^{\prime }=\Phi \wedge H_{0}\wedge H_{1}\wedge \ldots \wedge H_{mk-1}\wedge \Psi$.

Obviously, $x_{i}$ and $y_{j,i}$ are forced to be true for i=1,2,…,k,j=1,2,…,mk (this ensures that Ψ is satisfiable). In these forced variables, $x_{k},y_{1,k},y_{2,k},\ldots ,y_{mk-1,k}$ and $y_{mk,1},y_{mk,2},\ldots ,y_{mk,k}$ occur exactly in one clause of $\Phi^{\prime }$. Except for the (m+1)k variables, every variable occurs in exactly *s* clauses, and the absolute value of the difference between positive and negative occurrences of every variable is at most *d*. The number of unforced variables in $\Phi^{\prime }$ is still *n*. So $\Phi^{\prime }$ is a (m+1)k-forced-once *d*-regular (k,s)-CNF formula with *n* unforced variables.

Next, we will prove that the construction of Ψ is feasible. We focus on the satisfiability of the condition ii.

The variables of Ψ consists of two parts: *X* and *Y*. The variable set *Y* has (mk−1)(k−1) variables. The variable set *X* has k−1 variables. Every variable of *X* and *Y* occurs in exactly s−k−1 clauses of Ψ. Obviously, the number of literals of Ψ is (mk−1)(k−1)(s−k−1)+(k−1)(s−k−1). The number of clauses of Ψ is

$$\begin{array}{cc} \#cl(\Psi ) & =\frac{(mk-1)(k-1)(s-k-1)+(k-1)(s-k-1)}{k}\\ \; & =m(k-1)(s-k-1). \end{array}$$

When s≤2k, all literals in Ψ are positive literal and must satisfy the condition iii. When s>2k, the number of positive occurrences of the variables in Ψ is

$$\begin{array}{cc} pos(\Psi ) & =\left(mk-1\right)\left(k-1\right)\left(\left\lceil s/2\right\rceil -1\right)+\left(k-1\right)\left(\left\lceil s/2\right\rceil -1\right)\\ \; & =mk\left(k-1\right)\left(\left\lceil s/2\right\rceil -1\right). \end{array}$$

For k≥3,

$$\begin{array}{cc} mk\left(k-1\right)\left(\left\lceil s/2\right\rceil -1\right) & =m\left(k-1\right)\left(k\left\lceil s/2\right\rceil -k\right)\geq m\left(k-1\right)\left(3\left\lceil s/2\right\rceil -k\right)\\ \; & \geq m(k-1)(3s/2-k)>m(k-1)(s-k)\\ \; & >m(k-1)(s-k-1). \end{array}$$

So pos(Ψ)>#cl(Ψ). That indicates that the number of positive literals is more than that of clauses. That is, we can arrange a positive literal for every clause of Ψ, then randomly arrange other literals. Hence, Ψ can be constructed. □

**Theorem** **2.** *For k≥3 and s≥f(k,d)+1 and (s+d)/2>k−1, there exists a uniquely satisfiable d-regular (k,s)-CNF formula.*

**Proof.**  We will show a way to construct a uniquely satisfiable *d*-regular (k,s)-CNF formula.

By Lemma 3 and Lemma 4, for k≥3, s≥f(k,d)+1, (s+d)/2>k−1 and m≥1, we can construct a (m+1)k-forced-once *d*-regular (k,s)-CNF formula Ψ. It is assumed that the (m+1)k forced variables which occur only once are $X=\{x_{1},x_{2},\ldots ,x_{(m+1)k}\}$. Without loss of generality, we assume that forcing *n* of unforced variables to be true can turn Ψ into a uniquely satisfiable formula. Let $Y=\{y_{1},y_{2},\ldots ,y_{n}\}$ denote the *n* unforced variables. Let $t=\left\lceil n/(k-1)\right\rceil$. Constructing a uniquely satisfiable *d*-regular (k,s)-CNF formula is based on four stages, which are described as follows.

**Step 1** Divide the variables $y_{1},y_{2},\ldots ,y_{n}$ arbitrarily into *t* variable sets $Y_{1},Y_{2},\ldots ,Y_{t}$ of size k−1. Some variables of Ψ forced to be true are added, so that every variable set contains exactly k−1 variables (a variable forced to be false can be transformed to a variable forced to be true by flipping all occurrences of the variable). The variables $x_{1},x_{2},\ldots ,x_{(m+1)k}$ are arbitrarily divided into 4t+1 variable sets $X_{1},X_{2},\ldots ,X_{4t+1}$. Moreover, it should be guaranteed that any one of $X_{1},X_{2},\ldots ,X_{3t}$ has k−2 variables, any one of $X_{3t+1},\ldots ,X_{4t}$ has k−1 variables and $X_{4t+1}$ includes the rest. When *m* is appropriately chosen, the partition is feasible. Now assume $X_{4t+1}$ contains *r* variables.

**Step 2** For each 1≤i≤t, we will construct a formula $H_{i}$ using the variable sets $Y_{i},X_{i},X_{t+i},X_{2t+i}$ and $X_{3t+i}$.

For simplicity, let $Y_{i}=\left\{y_{1},\ldots ,y_{k-1}\right\}$, $X_{i}=\left\{x_{1},\ldots ,x_{k-2}\right\}$, $X_{t+i}=\left\{x_{t+1},\ldots ,x_{t+k-2}\right\}$, $X_{2t+i}=\left\{x_{2t+1},\ldots ,x_{2t+k-2}\right\}$ and $X_{3t+i}=\left\{x_{3t+1},\ldots ,x_{3t+k-1}\right\}$. For each 1≤i≤t, we introduce a new boolean variable set $Z_{i}=\left\{z_{1,0},z_{1,1},z_{2,0},z_{2,1},\ldots ,z_{k-1,0},z_{k-1,1}\right\}$ which does not occur in Ψ and perform the following steps to construct $H_{i}$.

(i) Let $z_{j,0}$ replace any one of positive occurrences of $y_{j}$, and $\neg z_{j,1}$ replace any one of negative occurrences of $y_{j}$ in Ψ, for j=1,2,…,k−1. If $y_{j}$ does not occur as a positive literal, then we let $z_{j,0}$ replace one of other negative occurrences of $y_{j}$ in Ψ and flip all occurrences of $z_{j,0}$ in the following formulas $H_{i1},H_{i2},H_{i3},H_{i4}$. If $y_{j}$ does not occur as a negative literal, then we perform similar operations.
(ii) Let
  $$\begin{array}{cc} H_{i1} & =\wedge_{j=1}^{k-1}\left(y_{j}\vee \neg z_{j,0}\vee \neg x_{1}\vee \ldots \vee \neg x_{k-2}\right),\\ H_{i2} & =\wedge_{j=1}^{k-1}\left(z_{j,0}\vee \neg z_{j,1}\vee \neg x_{t+1}\vee \ldots \vee \neg x_{t+k-2}\right),\\ H_{i3} & =\wedge_{j=1}^{k-1}\left(z_{j,1}\vee \neg y_{j}\vee \neg x_{2t+1}\vee \ldots \vee \neg x_{2t+k-2}\right),\\ H_{i4} & =\wedge_{j=1}^{k-1}\left(z_{j,1}\vee \neg x_{3t+1}\vee \ldots \vee \neg x_{3t+k-1}\right). \end{array}$$

Define $H_{i}=H_{i1}\wedge H_{i2}\wedge H_{i3}\wedge H_{i4}$. The new formula with all substitutions performed on Ψ is denoted as $\Psi_{1}$.

**Step 3** We will make up the gap of the number of occurrences of every variable. Using the variables in sets *X* and $Z=\{Z_{i},i=1,2,\ldots ,t\}$, we construct a formula $\Psi_{2}$ that satisfies the following conditions.

(i) For i=1,…,t, each $z_{j,0},j=1,2,\ldots ,k-1$ in the variable set $Z_{i}$ occurs in exactly s−3 clause of $\Psi_{2}$ and $pos\left(\Psi_{2},z_{j,0}\right)+1-neg\left(\Psi_{2},z_{j,0}\right)=min\left(d,1\right)$.
(ii) For i=1,…,t, each $z_{j,1},j=1,2,\ldots ,k-1$ in the variable set $Z_{i}$ occurs in exactly s−4 clauses of $\Psi_{2}$ and $pos\left(\Psi_{2},z_{j,1}\right)-neg\left(\Psi_{2},z_{j,1}\right)=min\left(d,1\right)$.
(iii) Each variable *x* in $X_{1},X_{2},\ldots ,X_{4t}$ occurs in exactly s−k clauses of $\Psi_{2}$,
  $$pos(\Psi_{2},x)=s-k\;\mathrm{for}\;s<2k\;\mathrm{or}\;pos\left(\Psi_{2},x\right)=\left\lceil s/2\right\rceil -1\;\mathrm{for}s\geq 2k.$$
(iv) Each variable *x* in $X_{4t+1}$ occurs in exactly s−1 clauses of $\Psi_{2}$ and
  $$pos\left(\Psi_{2},x\right)+1-neg\left(\Psi_{2},x\right)=min\left(d,1\right).$$
(v) Every clause of $\Psi_{2}$ must have at least one positive occurrence of any one of the variables.

**Step 4** Let $\Phi =\Psi_{1}\wedge \left(\wedge_{i=1}^{t}H_{i}\right)\wedge \Psi_{2}$.

Clearly, Φ is a *d*-regular (k,s)-CNF formula. All variables in the set *X* are forced to be true. Hence, $z_{j,1},j=1,2,\ldots ,k-1$ is forced to be true by $H_{i4}$. By Lemma 2, $z_{j,0},z_{j,1}$ and $y_{j}$ are forced to be the same value. Given that, every variable in *Y* and *Z* is forced to be true, too. Because all variables in *X* and *Z* are forced to be true, $\Psi_{2}$ is apparently satisfiable. Thus, it can be concluded that Φ has only forced variables and the unique solution. That is, Φ is a uniquely satisfiable *d*-regular (k,s)-CNF formula.

Next, we will discuss the feasibility of constructing Φ. We focus on the formula $\Psi_{2}$. For $\Psi_{2}$, the number of positive literals should be more than that of clauses.

The variable set *Z* generates t(k−1)(s−3)+t(k−1)(s−4) literals in $\Psi_{2}$. The variable set *X* generate 3t(k−2)(s−k)+t(k−1)(s−k)+r(s−1) literals in $\Psi_{2}$. The number of clauses of $\Psi_{2}$ is

$$\#cl\left(\Psi_{2}\right)=\frac{t(k-1)(2s-7)+3t(k-2)(s-k)+t(k-1)(s-k)+r(s-1)}{k}.$$

Every variable of *Z* generates $\left\lceil s/2\right\rceil -2$ positive literals in $\Psi_{2}$, and very variable of $X_{4t+1}$ generates $\left\lceil s/2\right\rceil -1$ positive literals in $\Psi_{2}$. About the number of positive literals of $\Psi_{2}$, there are two situations. When s<2k, the number of positive literals of $\Psi_{2}$ is

$$pos\left(\Psi_{2}\right)=t\left(k-1\right)\left(2\left\lceil s/2\right\rceil -4\right)+3t\left(k-2\right)\left(s-k\right)+t\left(k-1\right)\left(s-k-1\right)+r\left(\left\lceil s/2\right\rceil -1\right).$$

When s≥2k, the number of positive literals of $\Psi_{2}$ is

$$pos\left(\Psi_{2}\right)=t\left(k-1\right)\left(2\left\lceil s/2\right\rceil -4\right)+3t\left(k-2\right)\left(\left\lceil s/2\right\rceil -1\right)+t\left(k-1\right)\left(\left\lceil s/2\right\rceil -1\right)+r\left(\left\lceil s/2\right\rceil -1\right).$$

Since k≥3 and s>k, we get $pos\left(\Psi_{2}\right)>\#cl\left(\Psi_{2}\right)$. To construct $\Psi_{2}$, We first arrange a positive literal for every clause, then randomly arrange other literals. That is, $\Psi_{2}$ can be constructed in polynomial time.

$\Psi_{1}$ and $H_{i}$ can obviously be constructed in polynomial time. Therefore, we can construct a uniquely satisfiable *d*-regular (k,s)-CNF formula Φ in polynomial time. □

In the previous proof, we construct a uniquely satisfiable *d*-regular (k,s)-CNF formula Φ by using a (m+1)*k*-forced-once *d*-regular (k,s)-CNF formula Ψ. *m* determines the number of forced variables of Ψ that only occurs once. If let *m* be 1 more than our demand, then Φ can preserve *k* forced variables that only occurs once. Therefore, we get the following lemma.

**Lemma** **5.** *For k≥3, s≥f(k,d)+1 and (s+d)/2>k−1, there exists a k-forced-once d-regular (k,s)-CNF formula *Ψ* where every variable is forced.*

**Lemma** **6.** *For k≥7, if a d-regular (k,s)-CNF formula is unsatisfiable then (s+d)/2>k−1.*

**Proof.**  By 13≤f(7)≤17 in [22], if a (7,*s*)-CNF formula is unsatisfiable, then s≥14. That is, for any integer d≥0, if a *d*-regular (7,*s*)-CNF formula is unsatisfiable, then s≥14. It implies that for k=7, if a *d*-regular (k,s)-CNF formula is unsatisfiable, we can obtain that (s+d)/2≥14/2>k−1.

By 24≤f(8)≤29 in [22], all (8,24)-CNF formulas are satisfiable. That is, for any integer d≥0, if a *d*-regular (8,*s*)-CNF formula is unsatisfiable, then s>24. As for k=8, if a *d*-regular (k,s)-CNF formula is unsatisfiable, we get (s+d)/2>k−1 again.

Using Lemma 1, all (8+r,24+3×r)-CNF formulas are satisfiable for any nonnegative integer *r*. That is to say, if a (8+r,s)-CNF formula is unsatisfiable, then s>24+3×r for any nonnegative integer *r*. For (24+3×r)/2>8+r−1, we obtain that for k≥7 if a (k,s)-CNF formula is unsatisfiable, then (s+d)/2>k−1. □

**Theorem** **3.** *For all k≥7 and s≥f(k,d)+1, there exist uniquely satisfiable d-regular (k,s)-CNF formulas.*

**Proof.**  By the definition of f(k,d), if k≥7 and s≥f(k,d)+1, there exists an unsatisfiable *d*-regular (k,s)-CNF formula. Using Lemma 6, we get (s+d)/2>k−1. By Theorem 2, we obtain that there exist uniquely satisfiable *d*-regular (k,s)-CNF formulas. □

By Theorem 3, for k≥7, we get u(k,d)≤f(k,d)+1. Matthews in [20] showed that f(k)≤u(k)≤f(k)+2. Using Theorem 2, it is easy to achieve f(k)≤u(k)≤f(k)+1.

**Theorem** **4.** *For all k≥3, f(k)≤u(k)≤f(k)+1.*

**Proof.**  Let *d* be a infinite integer. That is, any one of (k,s)-CNF formulas is a *d*-regular (k,s)-CNF formula and any one of *d*-regular (k,s)-CNF formulas is a (k,s)-CNF formula. It holds that f(k)=f(k,d) and (s+d)/2>k−1. Using Theorem 2, for a infinite integer *d*, k≥3 and s≥f(k)+1, there exists a uniquely satisfiable *d*-regular (k,s)-CNF formula. Obviously, a uniquely satisfiable *d*-regular (k,s)-CNF formula must be a uniquely satisfiable (k,s)-CNF formula. In other words, for k≥3 and s≥f(k)+1, there exists a uniquely satisfiable (k,s)-CNF formula. By f(k)≤u(k)≤f(k)+2 in [20], we obtain that k≥3, f(k)≤u(k)≤f(k)+1. □

**Corollary** **1.** *For k≥7 and s≥f(k,d)+1, there exists a k-forced-once d-regular (k,s)-CNF formula* Ψ *that has exactly one satisfying assignment.*

**Proof.**  The statement follows directly from Lemmas 5 and 6. □

## 5. A Parsimonious Polynomial Time Reduction

In [20], Matthews presented a parsimonious reduction from SAT to (k,s)-SAT for any k≥3 and s≥f(k)+2. We will transform parsimoniously a *k*-CNF formula into a *d*-regular (k,s)-CNF formula.

**Theorem** **5.** *For any constants k≥3, s≥f(k)+1 and (s+d)/2>k−1, there exists a parsimonious polynomial time reduction from k-CNF to d-regular (k,s)-CNF.*

**Proof.**  Let Ψ be an arbitrarily *k*-CNF formula. It is supposed that Ψ contains *m* clauses. Obviously, Ψ contains mk literals $L_{1,1},L_{1,2},\ldots ,L_{m,k}$. We will construct a *d*-regular (k,s)-CNF formula $\Psi^{\prime }$ that is SAT-equivalent with the formula Ψ, and they have the same number of solutions. Based on Lemma 5, we first construct a *k*-forced-once *d*-regular (k,s)-CNF formula Φ where every variable is forced. It is assumed that *k* forced variables that occur only once are $x_{1},x_{2},\ldots ,x_{k}$.

The reduction method has five steps, which are described as follows.

**Step 1** We introduce a new boolean variable set $Z=\{z_{i,j}:1\leq i\leq m,1\leq j\leq k\}$ to replace mk literals in Ψ in order to construct a new formula $\Psi_{1}$.

$$\Psi_{1}=\underset{1\leq i\leq m}{\wedge }\underset{1\leq j\leq k}{\vee }{L^{\prime }}_{i,j},\;\;{L^{\prime }}_{i,j}=\left\{\begin{array}{c} \;\;z_{i,j},\;if\;\;L_{i,j}=v\\ \neg z_{i,j},\;if\;\;L_{i,j}=\neg v \end{array}\right.,v\in \mathrm{var}\left(\Psi \right).$$

Here, $L_{i,j}$ is the *j*th literal of the *i*th clause of Ψ.

**Step 2** Let $\Phi_{i},1\leq i\leq m\left(k-1\right)$ be disjoint copies of the formula Φ with the variables $x_{j},1\leq j\leq k$ of Φ being renamed as $x_{i,j}$ in $\Phi_{i}$. All of $x_{i,j}$ are renumbered and formed a variable set $X=\{x_{i},1\leq i\leq mk\left(k-1\right)\}$. Let $\Psi_{2}=\wedge_{1\leq i\leq m(k-1)}\Phi_{i}$.

**Step 3** Let $\Psi_{3}=\wedge_{1\leq i\leq m,1\leq j\leq k}d_{i,j}$, and $d_{i,j}=z_{i,j}\vee \neg {z^{\prime }}_{i,j}\vee_{l=1}^{k-2}\neg x_{((i-1)m-j-1)(k-2)+l}$. Here $z_{i,j},z_{i,j}^{\prime }\in Z$ and if $z_{i,j}$ replaces a variable *v* in Ψ, then $z_{i,j}^{\prime }$ will point to the next variable in *Z* that replaces *v* (if $z_{i,j}$ is the last variable in *Z* that replaces *v*, then $z_{i,j}^{\prime }$ will point to the first variable in *Z* that replaces *v*). The variables in *Z* are sorted by their subscripts.

**Step 4** We construct a *k*-CNF formula $\Psi_{4}$ with two variable sets *X* and *Z*, satisfying the following conditions.

(i) Every variable $z_{i,j}$ of the variable set *Z* occurs in exactly s−3 clauses of $\Psi_{4}$, and if $z_{i,j}$ occurs negatively in $\Psi_{1}$,
  $$pos\left(\Psi_{4},z_{i,j}\right)-neg\left(\Psi_{4},z_{i,j}\right)=min\left(d,1\right).$$
  Otherwise
  $$neg\left(\Psi_{4},z_{i,j}\right)-pos\left(\Psi_{4},z_{i,j}\right)=min\left(d,1\right).$$
(ii) For 1≤i≤mk(k−2), every variable $x_{i}$ of *X* occurs in exactly s−2 clauses of the formula $\Psi_{4}$, and
  $$pos\left(\Psi_{4},x_{i}\right)-neg\left(\Psi_{4},x_{i}\right)=min\left(d,1\right).$$
(iii) For mk(k−2)+1≤i≤mk(k−1), every variable $x_{i}$ of the variable set *X* occurs in exactly s−1 clauses of the formula $\Psi_{4}$, and
  $$pos\left(\Psi_{4},x_{i}\right)+1-neg\left(\Psi_{4},x_{i}\right)=min\left(d,1\right).$$
(iv) Every clause of $\Psi_{4}$ must have at least one positive occurrence of any one of the variable set *X*.

**Step 5** We construct the formula $\Psi^{\prime }=\left\{\Psi_{1},\Psi_{2},\Psi_{3},\Psi_{4}\right\}$.

Obviously, every variable of $\Psi^{\prime }$ occurs in exactly *s* clauses, and the absolute value of the difference between positive and negative occurrences of every variable of $\Psi^{\prime }$ is at most *d*. Therefore, $\Psi^{\prime }$ is a *d*-regular (k,s)-CNF formula. Next, we will evaluate the feasibility of $\Psi^{\prime }$, SAT-equivalent with $\Psi^{\prime }$ and Ψ, the parsimony of the reduction.

First, we focus on the feasibility of $\Psi^{\prime }$. The formulas $\Psi_{1},\Psi_{2},\Psi_{3}$ apparently can be constructed in polynomial time. With respect to the formula $\Psi_{4}$, we need to consider the condition iv. That is to say, the number of positive occurrences of *X* in $\Psi_{4}$ should be more than the number of clauses of $\Psi_{4}$.

The variables of $\Psi_{4}$ consists of two parts: *X* and *Z*. The variable set *X* generates mk(k−2)(s−3)+mk(s−1) literals in $\Psi_{4}$. The variable set *Z* generates mk(s−3) literals in $\Psi_{4}$. The number of clauses of $\Psi_{4}$

$$\begin{array}{cc} \#cl(\Psi ) & =\frac{mk(s-3)+mk(k-2)(s-2)+mk(s-1)}{k}\\ \; & =m(s-3)+m(k-2)(s-2)+m(s-1)\\ \; & =m(k-2)(s-2)+m(2s-4). \end{array}$$

For 1≤i≤mk(k−2), $pos\left(\Psi_{4},x_{i}\right)-neg\left(\Psi_{4},x_{i}\right)=min\left(d,1\right)$ and $pos\left(\Psi_{4},x_{i}\right)+neg\left(\Psi_{4},x_{i}\right)=s-2$. So,

$$pos\left(\Psi_{4},x_{i}\right)=\left\lceil \left(s-2\right)/2\right\rceil =\left\lceil s/2\right\rceil -1.$$

For mk(k−2)+1≤i≤mk(k−1), $pos\left(\Psi_{4},x_{i}\right)+1-neg\left(\Psi_{4},x_{i}\right)=min\left(d,1\right)$ and $pos\left(\Psi_{4},x_{i}\right)+neg\left(\Psi_{4},x_{i}\right)=s-1$. So, $pos\left(\Psi_{4},x_{i}\right)+1=\left\lceil s/2\right\rceil$, $pos\left(\Psi_{4},x_{i}\right)=\left\lceil s/2\right\rceil -1$. The number of positive occurrences of *X* in $\Psi_{4}$

$$pos\left(\Psi_{4},X\right)=mk\left(k-2\right)\left(\left\lceil s/2\right\rceil -1\right)+mk\left(\left\lceil s/2\right\rceil -1\right).$$

For k≥3 and s>k, we get

$$\begin{array}{cc} pos(\Psi_{4},X) & >3m\left(k-2\right)\left(\left\lceil s/2\right\rceil -1\right)+3m\left(\left\lceil s/2\right\rceil -1\right)\\ \; & >m\left(k-2\right)\left(s-2\right)+m\left(k-2\right)\left(\left\lceil s/2\right\rceil -1\right)+3m\left(\left\lceil s/2\right\rceil -1\right)\\ \; & >m\left(k-2\right)\left(s-2\right)+4m\left(\left\lceil s/2\right\rceil -1\right)\\ \; & >m\left(k-2\right)\left(s-2\right)+m\left(2s-4\right)=\#cl\left(\Psi_{4}\right). \end{array}$$

Obviously, the number of positive literals of *X* is more than the number of clauses in $\Psi_{4}$. To construct $\Psi_{4}$, We first arrange a positive literal for every clause, then randomly arrange other literals. That is, the formula $\Psi_{4}$ can be constructed in polynomial time.

Second, we will prove that the formula Ψ is satisfiable if and only if the formula $\Psi^{\prime }$ is satisfiable.

It is assumed that Ψ is satisfied by a truth assignment τ on var(Ψ) and $\Phi_{i}$ is satisfied by a truth assignment $\tau_{i}$ on $var(\Phi_{i})$ for 1≤i≤m(k−1). Because $\Phi_{i}$ forces the variable $x_{i,j}$ to be true, $\tau_{i}\left(x_{i,j}\right)$ must be true. A truth assignment $\tau^{\prime }$ is defined by

$$\tau^{\prime }\left(v\right)=\left\{\begin{array}{cc} \tau (z), & if\;v\in var(\Psi_{1})\;and\;a\;variable\;z\;of\;var\;\Psi \;is\;replaced\;with\;v\\ \tau_{i}\left(v\right), & if\;v\in var(\Phi_{i}) \end{array}\right..$$

Obvious, the truth assignment $\tau^{\prime }$ can satisfy these formulas $\Psi_{1},\Psi_{2},\Psi_{3}$. Every clause of $\Psi_{4}$ must have at least one positive occurrence of any one of *X*. As a result, $\tau^{\prime }$ also can satisfy the formula $\Psi_{4}$. The formula $\Psi^{\prime }$ is a conjunction of $\Psi_{1},\Psi_{2},\Psi_{3},\Psi_{4}$. Thus, $\tau^{\prime }$ can satisfy the formula $\Psi^{\prime }$ certainly.

It is assumed that $\Psi^{\prime }$ is satisfied by a truth assignment τ over $var(\Psi^{\prime })$. Obviously, the truth assignment τ can satisfy these formulas $\Psi_{1},\Psi_{2},\Psi_{3},\Psi_{4}$. For $\Psi_{2}=\wedge_{1\leq i\leq m(k-1)}\Phi_{i}$, the truth assignment τ can satisfy these formulas $\Phi_{i},1\leq i\leq m\left(k-1\right)$. Because $\Phi_{i}$ forces the variable $x_{i,j}$ to be true,

(1) $$\tau (\neg x_{i,j})=false,1\leq i\leq mk,1\leq j\leq k-2.$$

We substitute Equation (1) into $\Psi_{3}$, and simplify $\Psi_{3}$. The simplified $\Psi_{3}$ contains some similar structure that are mentioned in Lemma 2. According to Lemma 2, if $z_{i}$ and $z_{j}$ replace the same variable of Ψ, $\tau \left(z_{i}\right)=\tau \left(z_{j}\right)$. Therefore, we define a truth assignment $\tau^{\prime }$ on var(Ψ) by

$$\tau^{\prime }\left(v\right)=\tau \left(z\right),\;\mathrm{if}\;a\;\mathrm{variable}\;v\;\mathrm{of}\;\Psi \;\mathrm{is}\;\mathrm{replaced}\;\mathrm{with}\;a\;\mathrm{variable}\;z\;\mathrm{in}\;\Psi_{1}.$$

Obviously, the truth assignment $\tau^{\prime }$ can satisfy the formula Ψ, and the formula Ψ is satisfiable.

Therefore, $\Psi^{\prime }$ is SAT-equivalent with Ψ.

Finally, we will explain why the polynomial-time reduction is parsimonious. If $\Psi^{\prime }$ is satisfiable, all variables in *X* are forced to be true. Due to the formula $\Psi_{3}$, all variables of *Z* that replaced the same variable of Ψ are forced to be the same value in every satisfying assignment. Thus, the number of satisfying assignments cannot be changed by introducing new variable set *Z*. Due to only one solution of Φ, $\Psi_{2}$ must not influence the number of satisfying assignments. Therefore, Ψ has as many satisfying assignments as the formula $\Psi^{\prime }$. □

## 6. Conclusions

For k≥3, *k*-SAT problem is a NP-complete problem. From Theorem 5, it demonstrates that there exists a polynomial time reduction from *k*-SAT to *d*-regular (k,s)-SAT for any constants k≥3, s≥f(k)+1 and (s+d)/2>k−1. That is to say, *d*-regular (k,s)-SAT problem is NP-complete in this case. For example, the 2-regular (3,4)-SAT problem is NP-complete. In other words, there exists a parsimonious polynomial time reduction from 3-CNF to 2-regular (3,4)-CNF. Although the parsimonious reduction does not increase the number of solutions, it adds numerous new variables to the original formula. That is, The new formula has bigger solution space than the original formula. It seems that the parsimonious reduction diluted these solutions and make the SAT problem harder to solve. This explains why a random regular (3,4)-SAT instance is satisfiable with high probability and can be easily solved, but the 2-regular (3,4)-SAT problem is NP-complete.

From Lemma 6, this suggests that for k≥7 and (s+d)/2<k, all *d*-regular (k,s)-CNF formulas are satisfiable. Consider a regular (k,s)-CNF formula *F* in which the positive and negative occurrences number of every variable do not exceed k−1. Obviously, s≤2k−2 and the formula *F* is a (2k−s−2)-regular (k,s)-CNF formula. For (s+d)/2=(s+2k−s−2)/2=k−1<k, we obtain that the formula *F* must be satisfiable for k≥7. That is, all regular (k,s)-CNF formulas in which the positive and negative occurrences number of every variable are less than *k*, can be satisfiable for k≥7. However, it is unknown whether this phenomenon exists for k<7.

We present the construction method of a uniquely satisfiable *d*-regular (k,s)-formula. Uniquely satisfiable *d*-regular (k,s)-SAT instances have their own characteristics. How to use uniquely satisfiable SAT instances to evaluate, analyze and improve some SAT solvers will be considered in the future.
